# Editorial: Producing, Sensing and Responding to Cellular Stress in Immunity

**DOI:** 10.3389/fimmu.2019.02053

**Published:** 2019-09-06

**Authors:** Heitor A. Paula-Neto, Renata M. Pereira, Leticia A. M. Carneiro

**Affiliations:** ^1^Faculdade de Farmácia, Universidade Federal do Rio de Janeiro, Rio de Janeiro, Brazil; ^2^Instituto de Microbiologia Paulo de Góes, Universidade Federal do Rio de Janeiro, Rio de Janeiro, Brazil

**Keywords:** stress response, homeostasis, immune response, host-pathogen, innate immunity, UPR, autphagy, integrated stress response

Stress, meaning any disturbance of the internal environment of a cell, can result not only from external stimuli but also from physiological processes such as the intrinsic free radical production by the metabolic functioning of mitochondria. Stressors can threaten the cell and therefore mechanisms were selected throughout evolution to cope with and adapt to cell stress. Since the immune system is, ultimately, a system to sense and respond to stress posed by tissue damage, cell injury, and/or pathogens, it is reasonable to assume that all those cell-autonomous pathways involved in stress response also play a key role in immunity. This *Frontiers in Immunology* Research Topic focuses on different stress responses and their role in host–pathogen interaction and immunity.

A clear example of intrinsic stress is given by the fundamental role of reactive oxygen species (ROS) in T-cell activation. Gnanaprakasam et al. discuss how T-cells use their antioxidant machinery to fine-tune ROS activity so that it is sufficient to activate and polarize T-cells, but well-controlled to not result in cell damage. Other non-infectious process that impacts immunity is cell death. Controlled forms of cell death are an ancestral mechanism involved in key aspects of the physiology of multicellular organisms, including the elimination of unwanted, damaged, or infected cells. Amarante-Mendes et al. provide an overview on the three major types of molecularly controlled forms of cells death—apoptosis, necroptosis, and pyroptosis—that participate in host defense through the elimination of infected cells. Furthermore, the authors discuss how these events are both regulated by signals derived from PRRs as well as a source of danger-associated molecular patterns (DAMPS) that trigger immune responses through PRRs.

The concept of DAMPs is also key to understand how self-molecules can alert the immune system that homeostasis has been compromised. Among well-known DAMPs are the chromatin-associated protein HMGB1, extracellular purine metabolites, and S100 proteins. S100 proteins are a family of cytosolic proteins with a plethora of functions in cellular homeostasis that, when released from the cell as a result of tissue damage or cellular stress, can serve as DAMPs. Xia et al. explore this aspect of S100 proteins and how it interferes with different steps of inflammatory responses including their functions as DAMPs, on macrophage migration and on tissue repair.

Apart from its role in physiological processes, such as the removal of dead cells, the immune system is well-recognized for its function in host defense, interaction with microbes, and immune surveillance. During an infection, it is critical for the host to properly assess the potential threat posed by a given pathogen. In this sense, stress response pathways can be instrumental in providing the cell with the ability to sense alterations on homeostasis and tissue damage caused during infections. Rodrigues et al. discuss how the highly conserved integrated stress response (ISR) can shape the host response to bacterial pathogens. By sensing alterations to cellular homeostasis, rather than the bacteria itself, the ISR initiates a cellular program that includes transcription of key genes, profound alterations in translation of new proteins, and cell-autonomous antimicrobial mechanisms, such as autophagy. Smith also discussed how cellular stress induced by invading pathogens (virus and bacteria) is sensed, focusing on the impacts in protein folding induced by infection. Unfolded protein response (UPR) contributes to host defense through cytokine induction. The downside of the enhancement of host response is that UPR response has been increasingly recognized in a variety of autoimmune and inflammatory diseases.

Although the host cell is partly prepared to induce pathways that intervene in infections by sensing changes in homeostasis, pathogens like *Leishmania* parasites can adapt to these pathways and even benefit from them. Vivarini et al. demonstrated that *Leishmania amazonensis* induces the activation of the transcriptional factor Nrf2 (Nuclear factor erythroid 2-related factor 2), a master regulator of phase II defense gene expression that protect cells from oxidative stress. The authors show that Nrf2 knockdown promotes oxidative stress and impairs parasite survival in macrophages. Using the combination of *in vitro, ex viv*o, and *in silico* approach, the group shows Nrf2/PKR cross-talk and reveals a central role of Nrf2 in human cutaneous leishmaniasis. Nrf2 activation by *L. amazonensis* also required PI3K/Akt signaling and autophagy mechanisms. Autophagy is also the focus of a review by Siqueira et al. This process, known as a cellular mechanism to recycle organelles or digest intracellular contents in times of energy shortage, also plays a key role in immunity against intracellular pathogens.

Besides its interaction with pathogenic microorganisms, the immune system is also involved in the interaction and control of commensal microbiota, which, in turn, play a major role in instructing the immune system and maintaining homeostasis. Dysbiosis, which is a disruption of the normal microbiota–host relationship, has been associated with a myriad of human diseases including metabolic disorders, autoimmunity, and cancer. Espinoza and Minamo compile evidence suggesting that dysbiosis triggers DNA damage response, either by producing genotoxins or by promoting chronic inflammation, leading to overexpression of NKG2D-L in stressed cells. Consequently, these cells are tagged to be eliminated by Natural Killer (NK) cells and various subsets of T cells, which could be linked to autoimmunity and carcinogenesis.

On the other hand, the ability of immune cells, in particular NK cells, to detect cells displaying signs of stress is crucial for tumor immunosurveillance. This has been used to design new therapies that not only have antiproliferative and cytotoxic effects but also boost antitumor immunity by rendering tumor cells better targets for NK cells. Zingoni et al. discuss how cellular stress pathways induced in various tumors by different chemotherapeutic regimens can stimulate NK cells' effector function and provide new therapeutic approaches.

This collection of review and original papers is an invitation for the reader to appreciate the view of the immune system as a platform designed for the sensing, detection, and response to stress (in a broad definition), that uses all the stress-coping machinery selected by evolution to ensure an appropriate interaction with environmental challenges and host survival.

## Author Contributions

All authors listed have made a substantial, direct and intellectual contribution to the work, and approved it for publication.

### Conflict of Interest Statement

The authors declare that the research was conducted in the absence of any commercial or financial relationships that could be construed as a potential conflict of interest.

